# Improvement of Fermented Fish Flour Quality Using Essential Oil Extracted From Fresh Leaves of *Pimenta racemosa* (Mill.) J. W. Moore

**DOI:** 10.1007/s13659-017-0132-z

**Published:** 2017-05-24

**Authors:** Euloge S. Adjou, René G. Dègnon, Edwige Dahouenon-Ahoussi, Mohamed M. Soumanou, Dominique C. K. Sohounhloue

**Affiliations:** Laboratory of Research and Study in Applied Chemistry, Polytechnic School of Abomey-Calavi, University of Abomey-Calavi, 01, P.O.B: 2009, Cotonou, Bénin

**Keywords:** Essential oil, *Pimenta racemosa*, *Galeoides decadactylus*, Fermented fish, Process, Benin

## Abstract

The aim of this study was to evaluate the efficacy of the essential oil extracted from fresh leaves of *Pimenta racemosa* in the improvement of fermented fish flour producing technology. Essential oil of *Pimenta racemosa* was extracted by hydrodistillation and its chemical composition was determined by GC and GC/MS. Different types of fermented fish flours from Lesser African Threadfin (*Galeoides decadactylus*) were produced by the modification of the traditional processing technology and the introduction of a step of essential oil adjunction during the process. Three different essential oil concentrations (0.5, 1.0 and 2.0 μL g^−1^) were investigated. Physicochemical, microbiological and nutritional analyzes were performed in order to evaluate the quality of the fermented fish flour produced. Results obtained revealed that the essential oil of *Pimenta racemosa* investigated has a chemical composition characterized by the presence of myrcene (25.1%), chavicol (7.5%) and eugenol (51.1%). Fermented fish flour produced have a good nutritional potential. However, on the microbiological level, only samples produced by adjunction of essential oil have a low level of microbial contamination, with an absence of pathogenic microorganisms.

## Introduction

The lack of animal protein in the human diet is one of the most characteristics of developing countries. This protein deficiency, particularly, affects children. In Republic of Benin, fishing has an important role in national socio-economic balance and contributes about 3% to gross domestic product (GDP) [1]. However, conservation of fish is became difficult due to the lack of adequate conservation system, and climatic and environmental conditions are favorable to its rapid degradation [2]. Among these fish species is *Galéoides decadactylus*, is a very nutritious fish, mainly marine and commonly used for the production of fermented fish in Benin [3]. To reduce these post-capture losses, several artisanal treatments are carried out. Among these, the fermentation of fish is nowadays one of the most conservation methods used. During the production, producers get losses of products because of unpleasant practices and uncontrolled phenomena [4]. Moreover, fermentation is an important method of processing and preserving, as it generally improves nutritional characteristics of fermented products. It is therefore associated with salting or drying, with the aim of reducing water activity and delaying or preventing the rapid proliferation of proteolytic and putrefactive bacteria [2]. In Benin the technique used to produce fermented fish is fermentation with salting and drying, and the product obtained is known as ‘Lanhouin’ [5]. It is widely used in southern Benin as flavor enhancers [2]. However, despite the very nutritious nature of these dried and salted fermented fish, several problems related to their hygienic quality as well as their suitability for conservation still remain [6]. According to CECAF (Fishery Committee for the Eastern Central Atlantic), over than one hundred species of fish lived in marine waters under Benin jurisdiction and fish biomass was estimated at over than 9660 tons of pelagic fish and about 6000 tons of demersal fish [3]. Among demersal fish species are the Lesser African Threadfin (*Galeoides decadactylus*). Its post-harvest losses are very important because of the very soft nature of its fleshes. Various treatments are performed to reduce these losses. The fermentation and drying to produce the“*lanhouin*” is one of the control strategies of loss reduction currently used in Benin [4]. However, a lot of problems on the microbiological quality of this fermented fish was reported [3].

The restrictions imposed by international food organism on the use of chemical synthesis preservatives due to health and environmental risks [7] increasingly encourage the use of essential oils in food preservation [8]. Essential oils as well as derived compounds possess a wide range of activities of which, the antimicrobial activity is most studied [9, 10]. Their applications as preservatives in food or antiseptics and disinfectants have been widely investigated [11]. Several studies have revealed the antimicrobial and antioxidant properties of essential oils [12] which could be an effective alternative to the use of synthetic chemical preservatives in food conservation.

Plants belonging to Myrtaceae family like *Pimenta racemosa* have retained the attention of researchers, not only because of their high diversity and their distribution around the world, but also for their variable use in popular medicines to treat diseases [13]. In Benin, the leaves of this plant are used in culinary preparations where the species is known as “laurel leaf”. Thus the present study aims to investigate the efficacy of the essential oil extracted from the leaves of *Pimenta racemosa* in the improvement of producing technology and the nutritional and microbiological qualities of fermented fish flours produced.

## Results and Discussion

The yield of essential oils of *Pimenta racemosa* was 2.13%, (v/w) during hydrodistillation, and oil was yellow in colour. Chemical analysis by GC/MS of the components of the oil led to identification of 24 components (Table 1) representing 96.4%. The major components of the *Pimenta racemosa* oil were myrcène (25.1%), chavicol (7.5%) and eugenol (51.1%). High proportions of aromatic compounds (56.6%) followed by hydrocarbon monoterpenes (29.9%) were also identified in the essential oil. Table 2 presented the results of the physicochemical characteristics of the different groups of fermented fish flour produced. These results indicated that the moisture of fermented fish flour produced was between 13.26 ± 0.11 and 14.06 ± 0.01%, with a pH varying from 6.17 ± 0.02 to 6.22 ± 0.04, and an acidity between 1.16 ± 0.06 and 1.81 ± 0.05%. The results obtained during the evaluation of the nutritional characteristics of the different groups of fermented fish flour produced (Table 3) indicated that fermented fish flour have good nutritional potential, with a protein content varying from 22.96 ± 0.02% and 23.26 ± 0.11%. Fermented fish flours produced were also rich in minerals such as sodium (1.36 ± 0.09–1.46 ± 0.07%), potassium (0.48 ± 0.07–0.69 ± 0.03%), calcium (1.88 ± 0.09–2.04 ± 0.06%) magnesium (0.18 ± 0.02–0.24 ± 0.05%) and iron (0.31 ± 0.08–0.43 ± 0.07 mg/kg). Statistical analyzes revealed that there is no significant difference (p < 0.05) between samples analyzed. Results obtained during the evaluation of the microbiological characteristics of the different group of fermented fish flour (Table 4) indicated that the total bacteria count in analyzed sampled was between 10 × 10^1^ and 8.0 × 10^4^ ufc/g. Total coliform, feacal coliform, ASRspores, *Staphylococcus aureus*, yeast and mould are also detected, with the absence of *Salmonella*spp in all analyzed sampled. Statistical analyses indicated that there is significant difference (p < 0.05) between microbiological characteristic of samples produced by adjunction of essential oil of *Pimenta racemosa,* when compared to the control.

**Table 1:** Chemical composition of essential oil of *Pimenta racemosa* investigated

Components	Kovats index (KI)	Percentage (%)
α-pinène	940	0.4
octèn-3-ol	974	2.4
β-pinène	982	0.1
myrcène	993	25.1
α-terpinène	1018	0.1
p-cymène	1022	0.7
limonène	1034	3.0
1,8-cinéole	1041	2.7
(*E*)-β-ocimène	1043	0.2
γ-terpinène	1058	0.1
terpinolène	1089	0.2
linalol	1092	0.6
terpinène-4-ol	1178	0.8
α-terpinéol	1188	0.7
chavicol	1250	7.5
eugénol	1368	51.1
β-caryophyllène	1440	0.1
α-humulène	1489	0.1
(*E*,*E*)-α-farnesène	1502	0.2
δ-cadinène	1533	0.1
torréyol	1638	0.1
T-cadinol	1665	0.1
diterpène (M+=272)	1941	0.6
diterpène (M+=272)	1981	0.2
Total		96.4

**Table 2:** Physicochemical characteristics of different types of fermented fish flours investigated

Fermented fish flours	Moisture (%)	pH	Acidity (%)
A	13.26 ± 0.11a	6.38 ± 0.03a	1.47 ± 0.09a
B	13.83 ± 0.09a	6.31 ± 0.06a	1.29 ± 0.02a
C	13.41 ± 0.07a	6.22 ± 0.04a	1.16 ± 0.06a
D (Control)	14.06 ± 0.01a	6.17 ± 0.02a	1.81 ± 0.05a

Values are mean (n = 3) ± SE. The means followed by same letter in the same column are not significantly different according to ANOVA and Tukey’s multiple Comparison tests

**Table 3:** Nutritional characteristics of different types of fermented fish flours investigated

Fermented fish flours	Protein (%)	Ash (%)	Sodium (%)	Potassium (%)	Calcium (%)	Magnesium (%)	Iron (mg/kg)
A	23.16 ± 0.07a	24.17 ± 0.04a	1.46 ± 0.07a	0.69 ± 0.03a	2.04 ± 0.06a	0.22 ± 0.05a	0.43 ± 0.07a
B	23.12 ± 0.09a	24.03 ± 0.08a	1.39 ± 0.02a	0.57 ± 0.04a	1.88 ± 0.09a	0.18 ± 0.02a	0.37 ± 0.02a
C	22.96 ± 0.02a	24.88 ± 0.06a	1.36 ± 0.09a	0.48 ± 0.07a	1.96 ± 0.04a	0.20 ± 0.04a	0.31 ± 0.08a
D (Control)	23.26 ± 0.11a	24.67 ± 0.11a	1.44 ± 0.02a	0.62 ± 0.04a	2.01 ± 0.07a	0.24 ± 0.05a	0.42 ± 0.06a

Values are mean (n = 3) ± SE. The means followed by same letter in the same column are not significantly different according to ANOVA and Tukey’s multiple Comparison tests

**Table 4:** Microbiological characteristics of different types of fermented fish flours investigated

Fermented fish flours	Total bacteria count	Total coliform count	Faecal coliform count	ASR spores count	*Staphylococcus aureus* count	*Salmonella *spp	Yeast and mold count
A	2.0 × 10^1^	<10	<10	Absence	<10	Absence	<10
B	1.0 × 10^1^	<10	<10	Absence	<10	Absence	<10
C	2.0 × 10^1^	<10	<10	Absence	<10	Absence	<10
D (Control)	8.0 × 10^4^	2.0 × 10^2^	1.4 × 10^2^	2.0 × 10^1^	4.0 × 10^2^	Absence	3.0 × 10^2^

Fermentation has been used in many parts of world for the preparation of flavoured fish products which can add variety to the diet and contribute greatly to the general nutrition of large populations. Because of this, the development of such products is of great importance and a large number of traditional methods have evolved [14]. In Africa, salting and drying of fish for preservation is often accompanied by fermentation. But the period is short (a few days) and the product is not transformed into a paste or sauce. The products are all characterized by a strong odour do to the uncontrolled fermentation process. The characteristic smell of fermented fish is the result of enzymatic and microbiological activity in the fish muscle. These fermentation process was done by microorganisms. Indeed, fish in its natural environment has its own microflora in the slime on its body, in its gut and in its gills. These microorganisms, as well as the enzymes in the tissues of the fish, bring about putrefactive changes in fish when it dies. Furthermore, the microorganisms generally present in the salt used for salting also contribute to the degradative changes in the fish. There are halophile, halotolerant or osmophile microorganisms. Generally, halophile microrganisms grow optimally at high salt concentrations but are unable to grow in salt-free media. Halotolerant microorganisms grow best without significant amounts of salt but can also grow in concentrations higher than that of sea water. Osmophile microorganisms are those which can grow under high osmotic pressure. The combined action of the fermentative activity of these microorganisms in uncontrolled fermentation process contributes to the proliferation of undesirable and pathogenic microorganisms as highlighted by the present study (Table 4), and also reported by previous work [3].

The results obtained during the investigation of the microbiological characteristics of the fermented fish flours produced have indicated a significant reduction of microbial quantum in the fermented fish flours produced by the adjunction of essential oil. These results underlined the high antimicrobial potential of essential oil of *Pimenta racemosa*. Several studies have also reported the high antimicrobial potential of this essential oil. Indeed, Burt [15] reported that the essential oil of *Pimenta racemosa* exhibited strong antibacterial activity on *Escherichia coli* O 157: H7. The strong antimicrobial activity of this essential oil, could be due to the presence in the oil of the main component with high antimicrobial activity such as eugenol. Indeed, eugenol is a remarkably versatile molecule incorporated as a functional ingredient in numerous products and has found application in the pharmaceutical, agricultural, fragrance, flavour, cosmetic and various other industries. Its vast range of pharmacological activities has been well-researched and includes antimicrobial, anti-inflammatory, analgesic, anti-oxidant and anticancer activities. In addition, it is widely used in agricultural applications to protect foods from micro-organisms during storage. Singh [16] revealed that eugenol has inhibited the growth of Gram-positive (*Bacillus cereus*, *B. subtilis*, *Staphylococcus aureus*) and Gram-negative (*Escherichia coli*, *Salmonella typhi*, *Pseudomonas aeruginosa*) bacteria. According to the same authors, this inhibition was high in comparison to ampicillin (1 mg/mL) used as a positive control.

Several other studies have confirmed the antibacterial activity of eugenol against various pathogens such as *E. coli*, *B. cereus*, *Helicobacter pylori*, *S. aureus*, *S. epidermidis*, *Streptococcus pneumoniae* and *S. pyogenes* amongst numerous others [17, 18]. It is believed to have antibacterial property by the inhibition of extracellular enzymes synthesis and disruption of the cell wall structure resulting in lack of cytoplasm, cytoplasm granulation, cytoplasm hyperacidity, and depletion of intracellular ATP pool [19]. However, despite of the presence of eugenol in high proportion in this essential oil, its high antimicrobial potential could also be due to the synergic of action between main components and minority components of the essential oil.

## Experimental Sction

### Collection of Plant Leaves

Plant materials used for essential oil (EO) extraction were fresh leaves from *Pimenta racemosa*. Plants were collected at Abomey-calavi (southern Benin) and identified at the Benin national herbarium, where voucher specimens are deposited.

### Essential Oil Extraction

The EO tested was extracted by the hydro-distillation method using Clevenger-type apparatus. The oil recovered was dried over anhydrous sodium sulfate and stored at 4 °C until it was used [20].

### Gas Chromatography–Mass Spectrometry Analysis

The EO were analyzed by gas chromatograph (Perkin Elmer Auto XL GC; Waltham, MA, USA) equipped with a flame ionisation detector, and the GC conditions were EQUITY-5 column (60 m × 0.32 mm × 0.25 µm); H_2_ as the carrier gas; column head pressure 10 psi; oven temperature program isotherm 2 min at 70 °C, 3 °C/min gradient 250 °C, isotherm 10 min; injection temperature, 250 °C; detector temperature 280 °C. Gas chromatography–mass spectrometry (GC–MS) analysis was performed using a Perkin Elmer Turbomass GC–MS. The GC column was EQUITY-5 (60 m × 0.32 mm × 0.25 µm); fused silica capillary column. The GC conditions were injection temperature, 250 °C; column temperature, isothermal at 70 °C for 2 min, then programmed to 250 °C at 37 °C/min and held at this temperature for 10 min; ion source temperature, 250 °C. Helium was the carrier gas. The effluent of the GC column was introduced directly into the source of MS and spectra obtained in the EI mode with 70 eV ionisation energy. The sector mass analyzer was set to scan from 40 to 500 amu for 22 s. The identification of individual compounds is based on their retention times, retention indices relative to C_5_–C_18_ n-alkanes, and matching spectral peaks available in the published data [21].

### Production of Fermented Fish Flours

**Fig. 1:**
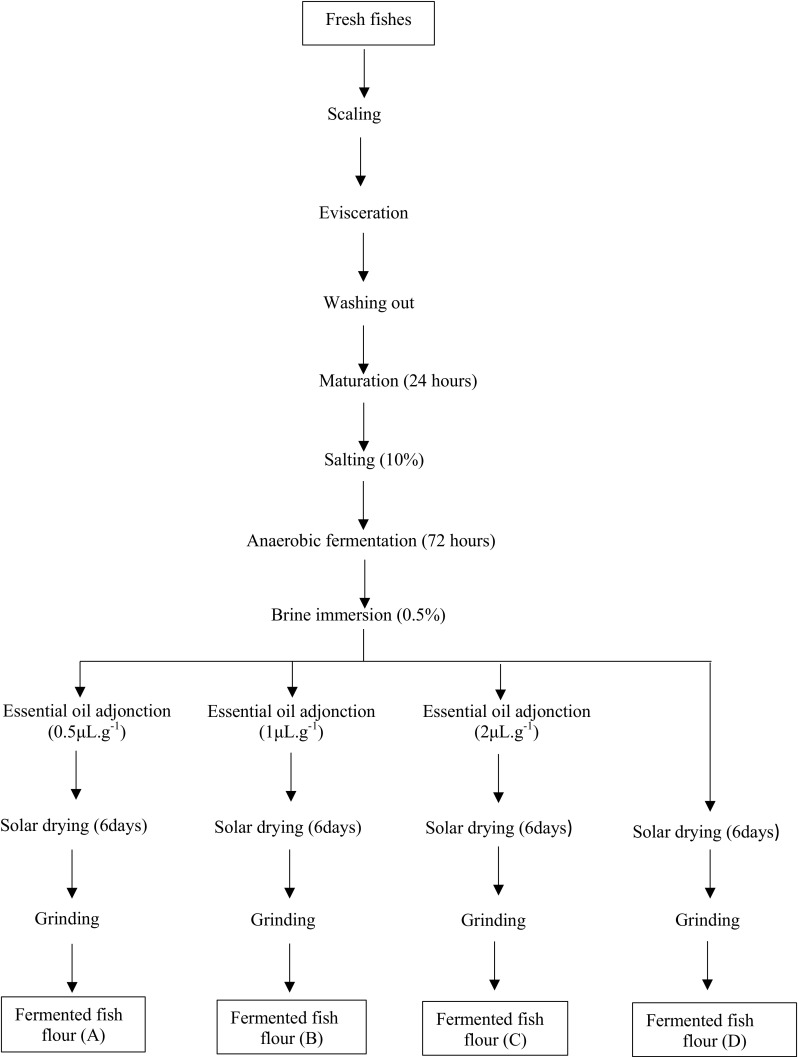
Process diagram of different fermented fish flours production

The different types of fermented fish flour used in this study were produced in the field of fermented fish production located in the Artisanal Fishing Port of Cotonou (southern Benin). The production methods used can be described as follows: samples of fresh fishes (*Galéoides decadactylus*) are scaled and eviscerated. After washing, it was left for 24 h for maturation. During this step, the fish is subjected to a tissue degradation process, under the action of enzymes and microorganisms. After the maturation step, fishes are salted by introducing the salt into the evisceration slot made under the operculum, and also into the gills and over the entire body of the fish. Fishes are now ready to the anaerobic fermentation process which duration is 3 days. After the anaerobic fermentation step, fish’s samples are then rinsed by immersion in brine (0.5%). The fish thus treated are then separated into four (4) parts labelled respectively A, B, C and D. The first three parts of fish (A, B, C) are treated with the essential oil of *Pimenta racemosa* by adjunction at doses of 0.5, 1.0 and 2.0 μL g^−1^. These doses were chosen because of the strong fragrance of the essential oil. The fourth part of fish’s samples (D) are not treated with the essential oil and is therefore considered as a control. Fishes are set to solar drying for 6 days according to traditional technology. After the solar drying step, fish’s samples are then ground using a conventional mechanical mill, which is found at the fermented fish production sites of the area’s study. Figure 1 indicated the technological diagram used for fermented fish flour production.

### Determination of Physicochemical Parameters

Moisture content of samples was determined by desiccation method [22]. A clean platinum dish was dried in an oven and cooled in a desiccator and weighed. From each sample, 5 g was weighed and spread on the dish, the dish containing the sample was weighed. It was then transferred into the air oven at 105 °C to dry until a constant weight was obtained and the loss in mass was determined. In order to obtain the pH of the samples, 5 g of each sample was weighed and suspended in 10 ml of distilled water. The pH was determined with a digital pH-meter (HANNA HI 98129). Acidity of samples was determined by titration with 0.01 mol/L of sodium hydroxide solution, using phenolphthalein as indicator [23].

### Nutritional Analysis

Protein was analyzed by the Microkjedhal nitrogen method, using a conversion factor of 6.25 and Ash was determined according to the standard methods described by the Association of Official Analytical Chemists [23]. Minerals were analyzed by dry-ashing 1 g of the sample at 550 °C in a furnace. The ash obtained was dissolved in 10% HCl, filtered with filter paper and made up to standard volume with deionised water. Flame photometer was used to determine sodium and potassium contents of the samples, while calcium, iron and magnesium contents were determined using atomic absorption spectrophotometer (Perkin Elmer, Model 403) [24].

### Microbiological Analysis

To 25 g of each sample, 225 ml of peptone water was added and homogenized. From the initial concentration, appropriate decimal dilutions were prepared and aliquots were plated in duplicates on various media. Plate count agar was used for the total bacterial count. Plates were incubated at 30 °C for 72 h. Desoxycholate was used for the total coliforms count and plates were incubated at 30 °C for 24 h. Desoxycholate was also used for the faecal coliforms count. In this case, plates were incubated at 44 °C and the identification was made using EMB (Eosine Methylene blue). Tryptone Sulfite Neomicin Agar was used for Anaerobic Sulfito Reducer (ASR) count and tubes were incubated at 37 °C for 24 h. *Staphylococcus aureus* count was performed as described by the standard NF EN ISO 6888-1. The method used for detection of *Salmonella* spp. is that specified by the standard NF V 08-052. After incubation, the number of colonies was tracked using a colony counter. The number of bacteria expressed as Colony Forming Units per gram (CFU/g) was then determined by calculation, considering the factors of dilution. The method used for detection of yeast and fungi in samples was performed using dilution plating method. 10 g of each sample were separately added to 90 ml of sterile water containing 0.1% peptone water. This was thoroughly mixed to obtain the 10^−1^ dilution. Further tenfold serial dilutions up to 10^−4^ were made. One milliliter of each dilution was separately placed in Petri dishes, over which 10–15 ml of Potato Dextrose Agar with 60 µg/ml of chloramphenicol (PDAC) was poured. The plates were incubated at 28 ± 2 °C for 7 days [25]. All media used for microbiological analysis were prepared as indicated by the manufacturer.

### Statistical Analyses

The data generated from these studies were analyzed using Statistical Analysis Software (SAS) and SYSTAT 5.05. The statistical analyses carried out were mean and standard deviation and analysis of variance (ANOVA) [26].

## Conclusion

This study underlined the nutritional potential of lesser African threadfin fermented flour and the importance of the use of essential oil of *Pimenta racemosa* in the improvement of the microbiological quality of this fermented product. Nevertheless, due to the possible interaction between the composition of fermented fish and the essential oil, further investigations are necessary to identify the conditions that maximize the activity of the essential oil, without detrimental effects on the organoleptic properties of the fermented food product.
